# New Therapeutic Options for Type 2 Diabetes Mellitus and Their Impact Against Ischemic Heart Disease

**DOI:** 10.3389/fphys.2022.904626

**Published:** 2022-06-27

**Authors:** Malak Almutairi, Jordan S. F. Chan, John R. Ussher

**Affiliations:** ^1^ Saudi Food and Drug Authority, Riyadh, Saudi Arabia; ^2^ Faculty of Pharmacy and Pharmaceutical Sciences, University of Alberta, Edmonton, AB, Canada; ^3^ Alberta Diabetes Institute, University of Alberta, Edmonton, AB, Canada; ^4^ Cardiovascular Research Centre, University of Alberta, Edmonton, AB, Canada

**Keywords:** type 2 diabetes mellitus, glucagon-like peptide-1 receptor agonists, sodium-glucose cotransporter-2 inhibitors, ischemic heart disease, myocardial infarction

## Abstract

Type 2 diabetes mellitus (T2DM) greatly increases risk for cardiovascular disease, including ischemic heart disease and myocardial infarction. With the completion of several cardiovascular outcomes trials (CVOTs) for new glucose-lowering therapies, including the sodium-glucose cotransporter-2 (SGLT2) inhibitors and glucagon-like peptide-1 receptor (GLP-1R) agonists, we now have strong evidence alluding to the cardioprotective nature of these agents in people with T2DM. These agents have frequently been observed to reduce rates for 3-point major adverse cardiovascular events, which encompass death from cardiovascular causes, nonfatal myocardial infarction, or nonfatal stroke. Herein we will provide an overview on whether reductions in nonfatal myocardial infarction and ischemic heart disease status are a key component of the improved cardiovascular outcomes in people with T2DM treated with either SGLT2 inhibitors or GLP-1R agonists. Observations from preclinical studies will be compared to their clinical counterparts, while being further interrogated to define potential mechanisms that may account for SGLT2 inhibitor or GLP-1R agonist-induced cardioprotection against ischemic heart disease. A better understanding of the role these agents have in impacting the progression of ischemic heart disease in individuals with T2DM will have a substantial impact in our management of this patient population.

## Introduction

Our society is experiencing an unfortunate explosion in the prevalence of obesity, which has greatly contributed to elevated rates of both type 2 diabetes mellitus (T2DM) and cardiovascular disease in our population. Cardiovascular disease in itself is a major cause of global mortality, with ischemic heart disease accounting for the majority of its disease burden. Ischemic heart disease is primarily attributed to atherosclerosis/coronary artery disease, which if allowed to progress or left untreated will manifest into myocardial infarction (MI) and ensuing ischemic cardiomyopathy/heart failure (Khan et al., 2020). Of importance, reports from the Framingham Heart Study have indicated that T2DM independently increases the risk of cardiovascular disease and mortality, primarily attributed to either MI and/or heart failure (Mahmood et al., 2014).

In light of this epidemiological data, it is vital that we improve our understanding of the mechanisms that precipitate diabetes-related heart disease, while using clinical evidence to best guide the therapeutic choices we use to manage cardiovascular health in highly susceptible diabetic individuals. Current evidence supports that tight glycemic control is strongly associated with reduced risk for microvascular complications (UK Prospective Diabetes Study (UKPDS) Group, 1998; Nathan et al., 2005). Conversely, well controlled glycemia in subjects with T2DM does not appear to reduce the risk for macrovascular cardiovascular disease such as ischemic heart disease and subsequent MI. Of relevance, the Action to Control Cardiovascular Risk in Diabetes (ACCORD) trial observed that intensive glucose lowering was associated with increased rates of death from cardiovascular causes (Action to Control Cardiovascular Risk in Diabetes Study, 2008). With health regulatory agencies (e.g. US Food and Drug Administration) now requiring pharmaceutical manufacturers to conduct cardiovascular outcomes trials (CVOTs) for new anti-diabetic therapies (Drucker and Goldfine, 2011), we now have access to cardiovascular outcomes data in thousands of subjects with T2DM treated with the latest therapies for T2DM. This includes the sodium-glucose co-transporter 2 (SGLT2) inhibitors and the incretin-based therapies, which includes both the glucagon-like peptide-1 (GLP-1) receptor (GLP-1R) agonists and the dipeptidyl peptidase 4 (DPP4) inhibitors. Accumulating evidence from these CVOTs has revealed much promise for GLP-1R agonists and SGLT2 inhibitors, but not DPP4 inhibitors, in terms of improving cardiovascular outcomes in people with T2DM.

The majority of these CVOTs are investigating major adverse cardiovascular events (MACE) as a primary endpoint, which is usually comprised of death from cardiovascular causes, nonfatal MI, or nonfatal stroke, referred to as 3-point MACE. Herein we will summarize the data from CVOTs relating to MI in the T2DM population treated with SGLT2 inhibitors or GLP-1R agonists, while interrogating preclinical data to delineate mechanisms that may account for either SGLT2 inhibitor or GLP-1R agonist mediated cardioprotection against ischemic heart disease. As animal models of chronic ischemia and subsequent MI are more reflective of the pathology of heart failure with reduced ejection fraction (Lindsey et al., 2021), we will focus specifically on studies of *in vivo* and *ex vivo* ischemia/reperfusion injury, which are more directly related to the nonfatal MI endpoint of 3-point MACE.

### The Pathology of Ischemic Heart Disease and Current Management

Ischemic heart disease is characterized by an imbalance between myocardial oxygen supply and demand. This often results from underlying atherosclerosis leading to inadequate perfusion of the myocardium, with endothelial dysfunction of the coronary arteries, myocardial hypoxia, and accumulation of metabolic by-products contributing to its pathology (Eisen et al., 2016). In the absence of restoration of coronary flow (i.e. reperfusion), the lack of oxygen/nutrient delivery to the myocardium prevents cardiomyocytes from generating the energy necessary to support contractile function, eventually resulting in cardiomyocyte death and loss of myocardial tissue (i.e. MI).

Accordingly, ischemic heart disease can be alleviated via surgical methods to improve myocardial perfusion (e.g. coronary artery bypass), mechanical means to improve coronary flow (e.g. coronary angioplasty), or pharmacologically to erode the coronary blockage with thrombolytics (e.g. streptokinase) (Smilowitz and Feit, 2016). In individuals with stable angina and/or ischemic heart disease, pharmacotherapy is primarily focused on either decreasing myocardial oxygen demand with *β*-blockers or calcium channel blockers, increasing myocardial oxygen supply with nitrates, or lowering circulating cholesterol with statins (Joshi and de Lemos, 2021). While individuals with T2DM and coexistent cardiovascular disease are often receiving concurrent treatment with many of the abovementioned therapies, it is imperative that we more critically evaluate how their respective glucose-lowering medications (e.g. SGLT2 inhibitors, GLP-1R agonists) also impact the progression of ischemic heart disease.

### SGLT2 Inhibitors and Cardiovascular Outcomes

The SGLT2 inhibitors (empagliflozin, canagliflozin, dapagliflozin, ertugliflozin) were developed as potential antidiabetic agents that promote glucose-lowering by blocking the reabsorption of renally filtered glucose, which is subsequently excreted in the urine (glucosuria) (Heerspink et al., 2016). While SGLT2 has been demonstrated to account for ∼97% of renally filtered glucose in normal physiology, treatment with SGLT2 inhibitors only prevents ∼50–60% of glucose reabsorption in practice, due to SGLT-1 compensating for glucose reabsorption in later regions of the proximal convoluted tubule, (Rieg et al., 2014; Heerspink et al., 2016). From a glucose-lowering mechanism of action viewpoint, SGLT2 inhibitors are unique in that they facilitate the elimination of glucose, rather than stimulate its uptake via either increasing insulin secretion or insulin sensitivity. As such, SGLT2 inhibitors have a notably lower risk of hypoglycemia, while lowering glycated hemoglobin by comparable levels versus other glucose-lowering medications (Heerspink et al., 2016).

Due to their favorable actions in CVOTs in combination with their safety profile and reasonable efficacy, the SGLT2 inhibitors have attained an increasing role in clinical practice guidelines (Davies et al., 2018; Diabetes Canada Clinical Practice Guidelines Expert et al., 2018). The salutary actions of large scale CVOTs involving SGLT2 inhibitors to reduce 3-point MACE have been extensively discussed and analyzed in previous reviews, though this benefit does not appear to be attributed to reductions in MI (Odutayo et al., 2021; Bhattarai et al., 2022; Razuk et al., 2022). For example, completion of the Empagliflozin Cardiovascular Outcome Event Trial in T2DM Patients (EMPA-REG) (Zinman et al., 2015) reported reductions in 3-point MACE in patients assigned empagliflozin compared to standard-of-care. However, rates of nonfatal MI or both nonfatal plus fatal MI were not the major reason behind this reduction in 3-point MACE, despite lower proportions of subjects receiving empagliflozin experiencing an event versus standard-of-care (4.5 versus 5.2% event rate; *p* = 0.22, 4.8 versus 5.4% event rate; *p* = 0.23). Likewise, the Canagliflozin Cardiovascular Assessment Study (CANVAS) also reported fewer events in subjects treated with canagliflozin using 3-point MACE (Neal et al., 2017). Rates of nonfatal MI were once again not significantly reduced for canagliflozin, but similar to empagliflozin did trend towards the right direction with regards to possible benefit (9.7 versus 11.6 events per 1000 patient-years; hazard ratio 0.85, 95% confidence interval 0.69–1.05.). On the contrary, the Dapagliflozin Effect on CardiovascuLAR Events (DECLARE-TIMI 58) trial only observed a trend to a reduction in events using 3-point MACE (*p* = 0.17) in dapagliflozin treated subjects with T2DM (Wiviott et al., 2019). Nonetheless, rates of MI did once more point towards a direction for improvement (11.7 versus 13.2 events per 1000 patient-years; hazard ratio 0.89, 95% confidence interval 0.77–1.01.). Most recently, findings from the Evaluation of Ertugliflozin Efficacy and Safety Cardiovascular Outcomes Trial (VERTIS-CV) have been the least promising for all SGLT2 inhibitor CVOTs, as ertugliflozin did not decrease 3-point MACE, with rates of fatal or nonfatal MI being unaffected versus standard-of-care (6.0 versus 5.8% event rate) (Cannon et al., 2020).

### SGLT2 Inhibitors in Preclinical Studies of Ischemic Heart Disease & Possible Mediators of Cardioprotection

While reductions in MI do not appear to be primarily involved in how SGLT2 inhibitors improve cardiovascular outcomes in people with T2DM, a plethora of preclinical studies have demonstrated that SGLT2 inhibitors decrease infarct size and alleviate ischemia/reperfusion injury. These salutary actions are observed regardless of whether the animals are nondiabetic or have T2DM, and appear to be a drug-class effect as benefit has been observed with empagliflozin, canagliflozin, and dapagliflozin (Sayour et al., 2021). For example, 12-week-old male nondiabetic C57BL/6J mice subjected to temporary left anterior descending (LAD) coronary artery occlusion for 45-min and 24-h of reperfusion, demonstrate reductions in infarct size and improved systolic function if pretreated with empagliflozin (10 mg/kg) once daily for 3-days prior to surgery (Lu et al., 2020). Of interest, this cardioprotection was attenuated if the mice were also pretreated with compound C for 3-days prior to surgery, suggesting that empagliflozin mediates the reduction in infarct size via stimulation of 5′AMP activated protein kinase (AMPK) activity. Similarly, pretreatment of male nondiabetic Sprague Dawley rats (250–300 g) for 7-days with empagliflozin (30 mg/kg) also decreased infarct size following temporary LAD coronary artery occlusion followed by 2-h of reperfusion (Seefeldt et al., 2021). Moreover, 12-week-old male nondiabetic C57BL/6J mice orally administered empagliflozin (10 mg/kg) for 6-weeks also exhibited reductions in infarct size following LAD coronary artery occlusion for 30-min and 2-h of reperfusion, but this time increased signal transducer and activator of transcription-3 rather than AMPK activity (Nikolaou et al., 2021).

In both Zucker lean and Zucker diabetic fatty rats, canagliflozin administration in the diet (166.7 mg/kg chow for ZL rats; 100 mg/kg chow for ZDF rats) for 4-weeks decreased infarct size during *ex vivo* Langendorff heart perfusions involving 35-min regional ischemia (*via* LAD coronary artery occlusion) and 2-h reperfusion (Lim et al., 2019). Furthermore, dapagliflozin treatment (40 mg/kg) once daily for 7-days prior to LAD coronary artery occlusion (30-min) followed by reperfusion (4-h) also decreased infarct size in male C57BL/6 mice and decreased markers of cardiac damage (circulating creatine kinase levels) (Yu et al., 2021). This cardioprotection requires autophagosome mediated degradation of NLR family pyrin domain containing 3 (NLRP3), as concurrent treatment with chloroquine to prevent lysosome-induced impairment of autophagosomes extinguished dapagliflozin’s ability to decrease NLRP3 levels and attenuate infarct size. It has also been suggested that SGLT2 inhibitors may induce cardioprotection by increasing circulating ketone bodies, and treatment of the isolated Langendorff perfused mouse heart with *β*-hydroxybutyrate (3 mM) improves functional recovery in response to 30-min global no flow ischemia and 40-min reperfusion (Byrne et al., 2020). Of interest, the NLRP3 inflammasome may also be implicated in this mechanism, as this *ex vivo* model of ischemia/reperfusion injury produced robust increases in myocardial NLRP3 expression that were also abolished by treatment with *β*-hydroxybutyrate (3 mM). Nevertheless, whether this explains SGLT2 inhibitor mediated cardioprotection against ischemia/reperfusion injury remains inconclusive.

Reasons for the discrepancy between the SGLT2 inhibitor CVOTs and preclinical studies could stem from the majority of preclinical data being performed in nondiabetic animals, despite some evidence of benefit in animals with T2DM. Furthermore, participants enrolled in CVOTs for glucose-lowering medications like SGLT2 inhibitors are often at high risk of cardiovascular disease or have existing cardiovascular disease. Hence, these individuals are often receiving cardiovascular therapies like *β*-blockers, which may make it more difficult to observe benefit, versus preclinical studies where the animals being subjected to experimental cardiovascular disease are not concurrently receiving approved cardiovascular therapies. While SGLT2 inhibitors appear to consistently decrease infarct size, it should also be noted that the benefit observed has often involved shorter reperfusion times that may ignore apoptotic death of cardiomyocytes, which may mask the actual infarct size with longer reperfusion times (>24 h) (De Villiers and Riley, 2020; Lindsey et al., 2021).

### GLP-1R Agonists and Cardiovascular Outcomes

GLP-1 is an incretin hormone secreted from enteroendocrine L-cells predominantly in the small intestine following nutrient ingestion (Campbell and Drucker, 2013). GLP-1 mediates its biological activity *via* the GLP-1R, a G-protein coupled receptor belonging to the Class B Family of G-protein coupled receptors. Following its initial identification in the pancreas, expression of the GLP-1R has been identified in numerous tissues including the lungs, enteric nervous system, regions of the brain, and of relevance to this specific review, in the heart, specifically the vascular smooth muscle cells and atrial cardiomyocytes (Campbell and Drucker, 2013). Regarding its glucose-lowering actions in T2DM, GLP-1 stimulates the islet *β*-cell GLP-1R to potentiate insulin secretion in a glucose-dependent manner, and thus the GLP-1R agonist drug class harbors low overall risk for hypoglycemia.

Similar to SGLT2 inhibitors, results from large-scale CVOTs have frequently demonstrated that GLP-1R agonists yield significant improvements in cardiovascular health in people with T2DM. However, the field was not met with initial excitement as both The Evaluation of Lixisenatide in Acute Coronary Syndrome (ELIXA) and Exenatide Study of Cardiovascular Event Lowering (EXSCEL) trials demonstrated that treatment of subjects with T2DM with lixisenatide or exenatide, respectively, were merely noninferior to placebo for 3-point MACE (Pfeffer et al., 2015; Holman et al., 2017). On the contrary, the majority of CVOTs for other GLP-1R agonists have been quite positive. This includes the Liraglutide Effect and Action in Diabetes: Evaluation of Cardiovascular Outcome Results (LEADER), the Trial to Evaluate Cardiovascular and Other Long-term Outcomes with Semaglutide (SUSTAIN-6), the Albiglutide and Cardiovascular Outcomes in Patients with T2D and Cardiovascular Disease (HARMONY Outcomes), and the Researching Cardiovascular Events With a Weekly Incretin in Diabetes (REWIND) CVOTs, all of which reported reductions in 3-point MACE (Marso et al., 2016a; Marso et al., 2016b; Hernandez et al., 2018; Gerstein et al., 2019). Unlike the SGLT2 inhibitor CVOTs, the improvement in 3-point MACE with numerous GLP-1R agonists does appear to involve attenuation of ischemic heart disease, as trends to decreased rates of nonfatal MI were observed in LEADER (6.0 versus 6.8% event rate; *p* = 0.11) and SUSTAIN-6 (2.9 versus 3.9% event rate; *p* = 0.12). Furthermore, event rates for nonfatal MI were significantly decreased in HARMONY Outcomes (4 versus 5% event rate; *p* = 0.03), though no changes were observed in REWIND (4.1 versus 4.3% event rate; *p* = 0.65).

Clinical studies in small numbers of subjects experiencing MI have also demonstrated favourable actions of GLP-1R agonists regarding relevant MI endpoints. For example, a 6-h exenatide infusion achieving a mean plasma concentration of 0.177 ± 0.069 nM administered 15-min prior to reperfusion onset in subjects undergoing coronary angioplasty to treat ST-segment elevation MI, reduced infarct size and increased the myocardial salvage index at ∼90-days post-infusion (Lonborg et al., 2012a; Lonborg et al., 2012b). Furthermore, in subjects with ST-segment elevation MI treated with exenatide (10 μg subcutaneous injection and 10 μg intravenous bolus) 5-min prior to coronary angioplasty, significant improvements in systolic function (ejection fraction) were observed 6-months later (Woo et al., 2013). In addition, subjects treated with exenatide also demonstrated reductions in markers of cardiac damage, which include circulating levels of creatine kinase-MB and troponin I. While these studies provide more support for GLP-1R agonists being cardioprotective in the setting of ischemic heart disease, the study populations are not equivalent to those enrolled in CVOTs of subjects with T2DM. Moreover, the EXSCEL CVOT did not observe improved outcomes against nonfatal MI for exenatide, though once weekly treatment with exenatide is also not comparable to exenatide infusion administered while undergoing angioplasty for a developing MI.

### GLP-1R Agonists in Preclinical Studies of Ischemic Heart Disease & Possible Mediators of Cardioprotection

Paralleling observations with SGLT2 inhibitors, GLP-1R agonists frequently induce protection against myocardial ischemia/reperfusion injury in preclinical models. The first observations of benefit were seen with intravenous infusion of native GLP-1 (4.8 pmol•kg^−1^•min^−1^) in male Sprague Dawley rats subjected to temporary LAD coronary artery occlusion for 30-min and followed by 2-h reperfusion, which markedly reduced infarct size (Bose et al., 2005), though concerns with short reperfusion times once again need to be considered. These salutary actions have been recapitulated with numerous GLP-1R agonists, including exenatide (10 μg 5-min prior to reperfusion followed by 10 μg twice daily for 3-days), which decreased infarct size following 75-min left circumflex coronary artery occlusion and 3-days reperfusion in Dalland Landrace pigs (sex not specified) (Timmers et al., 2009). Likewise, male Sprague-Dawley rats treated with albiglutide (1, 3, or 10 mg/kg once daily) for 3-days prior to 30-min LAD coronary artery occlusion and 24-h reperfusion also markedly reduced infarct size (Bao et al., 2011). Of interest, the albiglutide-induced protection against ischemia/reperfusion injury involved increases in myocardial glucose oxidation, which may be a key mechanism of GLP-1R agonist-induced cardioprotection. Increases in myocardial glucose oxidation have been directly shown to attenuate ischemia/reperfusion injury and decrease infarct size (Ussher et al., 2012). Using radioisotopes to measure flux through glucose oxidation in isolated working mouse hearts, the GLP-1R agonist liraglutide has been shown to only increase glucose oxidation rates if hearts are removed from mice that have been first treated systemically with liraglutide, but no increase is observed if the isolated working heart is directly treated with liraglutide (Almutairi et al., 2020). Such metabolic observations are consistent with liraglutide treatment (200 μg/kg) in C57BL/6J male mice improving functional recovery if their hearts are perfused in the Langendorff mode following 30-min global no flow ischemia and 40-min reperfusion, whereas no benefit was observed if the isolated heart was directly treated with liraglutide (30 nM) (Noyan-Ashraf et al., 2009).

GLP-1 and GLP-1R agonists may also decrease infarct size and mitigate ischemia/reperfusion injury by preventing cardiomyocyte apoptosis. Numerous markers of apoptosis, including terminal deoxynucleotidyl transferase dUTP nick end labeling and cleaved caspase 3 levels, as well as expression of pro-apoptotic/anti-apoptotic proteins (e.g. B-cell lymphoma 2 [Bcl2], Bcl-2 associated death promoter) often reflect decreased apoptosis (Bose et al., 2005; Noyan-Ashraf et al., 2009; Timmers et al., 2009; Bao et al., 2011). As the GLP-1R does not appear to be expressed in cardiomyocytes, at least in rodents (Kim et al., 2013), similar to what has been observed for the aforementioned GLP-1R agonist-induced changes in cardiac energy metabolism, it is likely that these anti-apoptotic actions are indirectly mediated. Increases in circulating insulin and decreases in circulating glucagon, respectively, could account for the stimulation of myocardial glucose oxidation and reduction in cardiomyocyte apoptosis (Drucker, 2016; Al Batran et al., 2018), though no study has extensively defined whether changes in the secretion of these hormones are responsible.

Caveolins, which are integral membrane proteins important for the formation of caveolae (membrane invaginations), play a key role in ischemia/reperfusion injury (Schilling et al., 2015), and evidence suggests that they may also contribute to GLP-1R agonist-induced reductions in infarct size. Male C57BL/6 mice (8–10 weeks of age) treated with exendin-4 (dose not specified) exhibited significant reductions in infarct size and circulating cardiac troponin I levels following 30-min LAD coronary artery occlusion and 2-h reperfusion (Tsutsumi et al., 2014). Intriguingly, these actions were completely abolished in male caveolin-3 deficient mice. These findings were reproduced in male C57BL/6 mice treated with exendin-4 (30 ng/kg intravenous infusion) immediately prior to 30-min LAD coronary artery occlusion and 2-h reperfusion, whereby increased migration of caveolin-3 to buoyant caveolar fractions was observed (Hamaguchi et al., 2015).

Another potential mechanism of GLP-1 mediated cardioprotection involves its DPP4 regulated cleavage product, GLP-1 (9–36). Although initially postulated to be biologically inert, a plethora of studies have demonstrated that GLP-1 (9–36) harbors cardiovascular actions. More specifically, direct treatment with GLP-1 (9–36) has been demonstrated to increase myocardial glucose uptake *ex vivo* (isolated Langendorff rat heart) and *in vivo* (canine) (Nikolaidis et al., 2005; Ban et al., 2008), while also inducing vasodilation in preconstricted (*via* phenylephrine) isolated mesenteric arteries. Furthermore, GLP-1 (9–36) can further be cleaved via neutral endopeptidase 24.11 to GLP-1 (28–36), which may act directly on mitochondria and decrease oxidative stress (Liu et al., 2012; Siraj et al., 2020), actions compatible with attenuated ischemia/reperfusion injury (Hausenloy and Yellon, 2013; Davidson et al., 2019). This proposed mechanism of cardioprotection is intriguing, as it could explain observations in studies where direct treatment of the isolated heart or *in vitro* cultures of cardiac myocytes lead to protective responses, despite GLP-1R expression being absent in cardiac myocytes (Ussher and Drucker, 2014). These observations may also explain why DPP4 inhibitors have had much less success regarding their actions in CVOTs, since DPP4 inhibitors prevent the formation of GLP-1 (9–36) and subsequently GLP-1 (28–36). However, the vast majority of GLP-1R agonists that have produced positive results in CVOTs are resistant to DPP4 mediated cleavage, questioning the relevance of this mechanism. Conversely, liraglutide is susceptible to DPP4 and neutral endopeptidase 24.11 mediated cleavage, leading to cleavage products distinct from that of GLP-1 (Malm-Erjefalt et al., 2010), and whether they are capable of producing similar actions to that of GLP-1 (9–36) and GLP-1 (28–36) is currently unknown.

Despite the preclinical and clinical studies for GLP-1R agonists being somewhat more aligned than that observed for SGLT2 inhibitors, the previous concerns described for the latter remain relevant here. This once again includes participants enrolled in CVOTs for GLP-1R agonists often receiving cardiovascular therapies like *β*-blockers, which may impact the overall translational impact of preclinical studies, whereas the preclinical studies have primarily studied nondiabetic animals.

### Summary Statement and Future Directions

Accumulating evidence from multiple CVOTs for SGLT2 inhibitors and GLP-1R agonists has resulted in major excitement in the fields of cardiovascular endocrinology and diabetic cardiology, as these agents display strong signs of cardioprotection in people with T2DM. While the primary endpoint in these CVOTs has been 3-point MACE, reductions in nonfatal/fatal MI and attenuated ischemic heart disease do not appear to be the primary driver of improved cardiovascular outcomes for SGLT2 inhibitors, but may play a more important role for the improved cardiovascular outcomes with GLP-1R agonists. Nonetheless, that does not necessarily imply that a GLP-1R agonist is the superior glucose-lowering agent versus an SGLT2 inhibitor for an individual with T2DM and coexisting ischemic heart disease. CVOTs for these agents are not consistent amongst their participant populations for cardiovascular risk/status, and there is a lack of direct comparisons between GLP-1R agonists and SGLT2 inhibitors in both clinical and preclinical studies. Advances in this area as these questions are answered will undoubtedly play a major role in therapeutic decision making with regards to managing both glycemia and ischemic heart disease in subjects with T2DM.

## References

[B17] Action to Control Cardiovascular Risk in Diabetes Study (2008). Effects of Intensive Glucose Lowering in Type 2 Diabetes. N. Engl. J. Med. 358, 2545–2559. 10.1056/NEJMoa0802743 18539917 PMC4551392

[B1] Al Batran R. Almutairi M. Ussher J. R. (2018). Glucagon-like Peptide-1 Receptor Mediated Control of Cardiac Energy Metabolism. Peptides 100, 94–100. 10.1016/j.peptides.2017.12.005 29412838

[B2] Almutairi M. Gopal K. Greenwell A. A. Young A. Gill R. Aburasayn H. (2020). The GLP-1R Agonist Liraglutide Increases Myocardial Glucose Oxidation Rates via Indirect Mechanisms and Mitigates Experimental Diabetic Cardiomyopathy. Can. J. Cardiol. 37 (1), 140–150. 10.1016/j.cjca.2020.02.098 32640211

[B3] Ban K. Noyan-Ashraf M. H. Hoefer J. Bolz S.-S. Drucker D. J. Husain M. (2008). Cardioprotective and Vasodilatory Actions of Glucagon-like Peptide 1 Receptor Are Mediated through Both Glucagon-like Peptide 1 Receptor-dependent and -independent Pathways. Circulation 117, 2340–2350. 10.1161/circulationaha.107.739938 18427132

[B4] Bao W. Aravindhan K. Alsaid H. Chendrimada T. Szapacs M. Citerone D. R. (2011). Albiglutide, a Long Lasting Glucagon-like Peptide-1 Analog, Protects the Rat Heart against Ischemia/reperfusion Injury: Evidence for Improving Cardiac Metabolic Efficiency. PLoS One 6, e23570. 10.1371/journal.pone.0023570 21887274 PMC3162574

[B5] Bhattarai M. Salih M. Regmi M. Al-Akchar M. Deshpande R. Niaz Z. (2022). Association of Sodium-Glucose Cotransporter 2 Inhibitors with Cardiovascular Outcomes in Patients with Type 2 Diabetes and Other Risk Factors for Cardiovascular Disease. JAMA Netw. Open 5, e2142078. 10.1001/jamanetworkopen.2021.42078 34985519 PMC8733833

[B6] Bose A. K. Mocanu M. M. Carr R. D. Brand C. L. Yellon D. M. (2005). Glucagon-like Peptide 1 Can Directly Protect the Heart against Ischemia/reperfusion Injury. Diabetes 54, 146–151. 10.2337/diabetes.54.1.146 15616022

[B7] Byrne N. J. Soni S. Takahara S. Ferdaoussi M. Al Batran R. Darwesh A. M. (2020). Chronically Elevating Circulating Ketones Can Reduce Cardiac Inflammation and Blunt the Development of Heart Failure. Circ. Heart Fail 13, e006573. 10.1161/CIRCHEARTFAILURE.119.006573 32493060

[B8] Campbell J. E. Drucker D. J. (2013). Pharmacology, Physiology, and Mechanisms of Incretin Hormone Action. Cell. Metab. 17, 819–837. 10.1016/j.cmet.2013.04.008 23684623

[B9] Cannon C. P. Pratley R. Dagogo-Jack S. Mancuso J. Huyck S. Masiukiewicz U. (2020). Cardiovascular Outcomes with Ertugliflozin in Type 2 Diabetes. N. Engl. J. Med. 383, 1425–1435. 10.1056/nejmoa2004967 32966714

[B10] Davidson S. M. Ferdinandy P. Andreadou I. Bøtker H. E. Heusch G. Ibáñez B. (2019). Multitarget Strategies to Reduce Myocardial Ischemia/Reperfusion Injury. J. Am. Coll. Cardiol. 73, 89–99. 10.1016/j.jacc.2018.09.086 30621955

[B11] Davies M. J. D’Alessio D. A. Fradkin J. Kernan W. N. Mathieu C. Mingrone G. (2018). Management of Hyperglycaemia in Type 2 Diabetes, 2018. A Consensus Report by the American Diabetes Association (ADA) and the European Association for the Study of Diabetes (EASD). Diabetologia 61, 2461–2498. 10.1007/s00125-018-4729-5 30288571

[B12] De Villiers C. Riley P. R. (2020). Mouse Models of Myocardial Infarction: Comparing Permanent Ligation and Ischaemia-Reperfusion. Dis. Model. Mech. 13, dmm046565. 10.1242/dmm.046565 33361140 PMC7687859

[B13] Drucker D. J. Goldfine A. B. (2011). Cardiovascular Safety and Diabetes Drug Development. Lancet 377, 977–979. 10.1016/s0140-6736(10)62299-4 21316097

[B14] Drucker D. J. (2016). The Cardiovascular Biology of Glucagon-like Peptide-1. Cell. Metab. 24, 15–30. 10.1016/j.cmet.2016.06.009 27345422

[B15] Eisen A. Giugliano R. P. Braunwald E. (2016). Updates on Acute Coronary Syndrome. JAMA Cardiol. 1, 718–730. 10.1001/jamacardio.2016.2049 27438381

[B16] Gerstein H. C. Colhoun H. M. Dagenais G. R. Diaz R. Lakshmanan M. Pais P. (2019). Dulaglutide and Cardiovascular Outcomes in Type 2 Diabetes (REWIND): a Double-Blind, Randomised Placebo-Controlled Trial. Lancet 394, 121–130. 10.1016/S0140-6736(19)31149-3 31189511

[B59] Hamaguchi E. Tanaka K. Tsutsumi R. Sakai Y. Fukuta K. Kasai A. (2015). Exendin-4, Glucagon-Like Peptide-1 Receptor Agonist, Enhances Isoflurane-Induced Preconditioning Against Myocardial Infarction *via* Caveolin-3 Expression. Eur. Rev. Med. Pharmacol. Sci. 19 (7), 1285–1290. 25912591

[B18] Hausenloy D. J. Yellon D. M. (2013). Myocardial Ischemia-Reperfusion Injury: a Neglected Therapeutic Target. J. Clin. Investig. 123, 92–100. 10.1172/jci62874 23281415 PMC3533275

[B19] Heerspink H. J. L. Perkins B. A. Fitchett D. H. Husain M. Cherney D. Z. I. (2016). Sodium Glucose Cotransporter 2 Inhibitors in the Treatment of Diabetes Mellitus. Circulation 134, 752–772. 10.1161/circulationaha.116.021887 27470878

[B20] Hernandez A. F. Green J. B. Janmohamed S. D'agostino R. B. Granger. Jones N. P. (2018). Albiglutide and Cardiovascular Outcomes in Patients with Type 2 Diabetes and Cardiovascular Disease (Harmony Outcomes): a Double-Blind, Randomised Placebo-Controlled Trial. Lancet 392, 1519–1529. 10.1016/S0140-6736(18)32261-X 30291013

[B21] Holman R. R. Bethel M. A. Mentz R. J. Thompson V. P. Lokhnygina Y. Buse J. B. (2017). Effects of Once-Weekly Exenatide on Cardiovascular Outcomes in Type 2 Diabetes. N. Engl. J. Med. 377, 1228–1239. 10.1056/nejmoa1612917 28910237 PMC9792409

[B22] Joshi P. H. De Lemos J. A. (2021). Diagnosis and Management of Stable Angina. JAMA 325, 1765–1778. 10.1001/jama.2021.1527 33944871

[B23] Khan M. A. Hashim M. J. Mustafa H. Baniyas M. Y. Al Suwaidi S. K. B. M. Alkatheeri R. (2020). Global Epidemiology of Ischemic Heart Disease: Results from the Global Burden of Disease Study. Cureus 12, e9349. 10.7759/cureus.9349 32742886 PMC7384703

[B24] Kim M. Platt M. J. Shibasaki T. Quaggin S. E. Backx P. H. Seino S. (2013). GLP-1 Receptor Activation and Epac2 Link Atrial Natriuretic Peptide Secretion to Control of Blood Pressure. Nat. Med. 19, 567–575. 10.1038/nm.3128 23542788

[B25] Lim V. G. Bell R. M. Arjun S. Kolatsi-Joannou M. Long D. A. Yellon D. M. (2019). SGLT2 Inhibitor, Canagliflozin, Attenuates Myocardial Infarction in the Diabetic and Nondiabetic Heart. JACC Basic Transl. Sci. 4, 15–26. 10.1016/j.jacbts.2018.10.002 30847415 PMC6390729

[B26] Lindsey M. L. Brunt K. R. Kirk J. A. Kleinbongard P. Calvert J. W. de Castro Brás L. E. (2021). Guidelines for *In Vivo* Mouse Models of Myocardial Infarction. Am. J. Physiology-Heart Circulatory Physiology 321, H1056–H1073. 10.1152/ajpheart.00459.2021 PMC8834230 34623181

[B27] Lipscombe L. Booth G. Butalia S. Dasgupta K. Eurich D. T. Goldenberg R. (2018). Erratum to "Pharmacologic Glycemic Management of Type 2 Diabetes in Adults": Canadian Journal of Diabetes 2018;42(S1):S88-S103. Can. J. Diabetes 42 (5), 575–S103. 10.1016/j.jcjd.2018.08.195 30268231

[B28] Liu Z. Stanojevic V. Brindamour L. J. Habener J. F. (2012). GLP1-derived Nonapeptide GLP1(28-36)amide Protects Pancreatic β-cells from Glucolipotoxicity. J. Endocrinol. 213, 143–154. 10.1530/joe-11-0328 22414687 PMC4096040

[B29] Lønborg J. Kelbæk H. Vejlstrup N. Bøtker H. E. Kim W. Y. Holmvang L. (2012a). Exenatide Reduces Final Infarct Size in Patients with ST-Segment-Elevation Myocardial Infarction and Short-Duration of Ischemia. Circ. Cardiovasc Interv. 5, 288–295. 10.1161/CIRCINTERVENTIONS.112.968388 22496084

[B30] Lønborg J. Vejlstrup N. Kelbæk H. Bøtker H. E. Kim W. Y. Mathiasen A. B. (2012b). Exenatide Reduces Reperfusion Injury in Patients with ST-Segment Elevation Myocardial Infarction. Eur. Heart J. 33, 1491–1499. 10.1093/eurheartj/ehr309 21920963

[B31] Lu Q. Liu J. Li X. Sun X. Zhang J. Ren D. (2020). Empagliflozin Attenuates Ischemia and Reperfusion Injury through LKB1/AMPK Signaling Pathway. Mol. Cell. Endocrinol. 501, 110642. 10.1016/j.mce.2019.110642 31759100

[B32] Mahmood S. S. Levy D. Vasan R. S. Wang T. J. (2014). The Framingham Heart Study and the Epidemiology of Cardiovascular Disease: a Historical Perspective. Lancet 383, 999–1008. 10.1016/s0140-6736(13)61752-3 24084292 PMC4159698

[B33] Malm-Erjefält M. Bjørnsdottir I. Vanggaard J. Helleberg H. Larsen U. Oosterhuis B. (2010). Metabolism and Excretion of the Once-Daily Human Glucagon-like Peptide-1 Analog Liraglutide in Healthy Male Subjects and its *In Vitro* Degradation by Dipeptidyl Peptidase IV and Neutral Endopeptidase. Drug Metab. Dispos. 38, 1944–1953. 10.1124/dmd.110.034066 20709939

[B34] Marso S. P. Bain S. C. Consoli A. Eliaschewitz F. G. Jódar E. Leiter L. A. (2016a). Semaglutide and Cardiovascular Outcomes in Patients with Type 2 Diabetes. N. Engl. J. Med. 375, 1834–1844. 10.1056/nejmoa1607141 27633186

[B35] Marso S. P. Daniels G. H. Brown-Frandsen K. Kristensen P. Mann J. F. E. Nauck M. A. (2016b). Liraglutide and Cardiovascular Outcomes in Type 2 Diabetes. N. Engl. J. Med. 375, 311–322. 10.1056/nejmoa1603827 27295427 PMC4985288

[B36] Nathan D. M. Cleary P. A. Backlund J. Y. Genuth S. M. Lachin J. M. Orchard T. J. (2005). Intensive Diabetes Treatment and Cardiovascular Disease in Patients with Type 1 Diabetes. N. Engl. J. Med. 353, 2643–2653. 10.1056/NEJMoa052187 16371630 PMC2637991

[B37] Neal B. Perkovic V. Mahaffey K. W. De Zeeuw D. Fulcher G. Erondu N. (2017). Canagliflozin and Cardiovascular and Renal Events in Type 2 Diabetes. N. Engl. J. Med. 377, 644–657. 10.1056/nejmoa1611925 28605608

[B38] Nikolaidis L. A. Elahi D. Shen Y.-T. Shannon R. P. (2005). Active Metabolite of GLP-1 Mediates Myocardial Glucose Uptake and Improves Left Ventricular Performance in Conscious Dogs with Dilated Cardiomyopathy. Am. J. Physiology-Heart Circulatory Physiology 289, H2401–H2408. 10.1152/ajpheart.00347.2005 16024574

[B39] Nikolaou P. E. Efentakis P. Abu Qourah F. Femminò S. Makridakis M. Kanaki Z. (2021). Chronic Empagliflozin Treatment Reduces Myocardial Infarct Size in Nondiabetic Mice through STAT-3-Mediated Protection on Microvascular Endothelial Cells and Reduction of Oxidative Stress. Antioxidants Redox Signal. 34, 551–571. 10.1089/ars.2019.7923 32295413

[B40] Noyan-Ashraf M. H. Momen M. A. Ban K. Sadi A.-M. Zhou Y.-Q. Riazi A. M. (2009). GLP-1R Agonist Liraglutide Activates Cytoprotective Pathways and Improves Outcomes after Experimental Myocardial Infarction in Mice. Diabetes 58, 975–983. 10.2337/db08-1193 19151200 PMC2661586

[B41] Odutayo A. Da Costa B. R. Pereira T. V. Garg V. Iskander S. Roble F. (2021). Sodium-Glucose Cotransporter 2 Inhibitors, All-Cause Mortality, and Cardiovascular Outcomes in Adults with Type 2 Diabetes: A Bayesian Meta-Analysis and Meta-Regression. J. Am. Heart Assoc. 10, e019918. 10.1161/jaha.120.019918 34514812 PMC8649541

[B42] Pfeffer M. A. Claggett B. Diaz R. Dickstein K. Gerstein H. C. Køber L. V. (2015). Lixisenatide in Patients with Type 2 Diabetes and Acute Coronary Syndrome. N. Engl. J. Med. 373, 2247–2257. 10.1056/nejmoa1509225 26630143

[B43] Razuk V. Chiarito M. Cao D. Nicolas J. Pivato C. A. Camaj A. (2022). SGLT-2 Inhibitors and Cardiovascular Outcomes in Patients with and without a History of Heart Failure: a Systematic Review and Meta-Analysis. Eur. Heart J. Cardiovasc Pharmacother. 11, pvac001. 10.1093/ehjcvp/pvac001 35021205

[B44] Rieg T. Masuda T. Gerasimova M. Mayoux E. Platt K. Powell D. R. (2014). Increase in SGLT1-Mediated Transport Explains Renal Glucose Reabsorption during Genetic and Pharmacological SGLT2 Inhibition in Euglycemia. Am. J. Physiology-Renal Physiology 306, F188–F193. 10.1152/ajprenal.00518.2013 PMC3920019 24226519

[B45] Sayour A. A. Celeng C. Oláh A. Ruppert M. Merkely B. Radovits T. (2021). Sodium-glucose Cotransporter 2 Inhibitors Reduce Myocardial Infarct Size in Preclinical Animal Models of Myocardial Ischaemia-Reperfusion Injury: a Meta-Analysis. Diabetologia 64, 737–748. 10.1007/s00125-020-05359-2 33483761 PMC7940278

[B46] Schilling J. M. Roth D. M. Patel H. H. (2015). Caveolins in Cardioprotection - Translatability and Mechanisms. Br. J. Pharmacol. 172, 2114–2125. 10.1111/bph.13009 25377989 PMC4386985

[B47] Seefeldt J. M. Lassen T. R. Hjortbak M. V. Jespersen N. R. Kvist F. Hansen J. (2021). Cardioprotective Effects of Empagliflozin after Ischemia and Reperfusion in Rats. Sci. Rep. 11, 9544. 10.1038/s41598-021-89149-9 33953281 PMC8100147

[B48] Siraj M. A. Mundil D. Beca S. Momen A. Shikatani E. A. Afroze T. (2020). Cardioprotective GLP-1 Metabolite Prevents Ischemic Cardiac Injury by Inhibiting Mitochondrial Trifunctional Protein-α. J. Clin. Investig. 130, 1392–1404. 10.1172/jci99934 31985487 PMC7269572

[B49] Smilowitz N. R. Feit F. (2016). The History of Primary Angioplasty and Stenting for Acute Myocardial Infarction. Curr. Cardiol. Rep. 18, 5. 10.1007/s11886-015-0681-x 26699632

[B50] Timmers L. Henriques J. P. S. De Kleijn D. P. V. Devries J. H. Kemperman H. Steendijk P. (2009). Exenatide Reduces Infarct Size and Improves Cardiac Function in a Porcine Model of Ischemia and Reperfusion Injury. J. Am. Coll. Cardiol. 53, 501–510. 10.1016/j.jacc.2008.10.033 19195607

[B51] Tsutsumi Y. M. Tsutsumi R. Hamaguchi E. Sakai Y. Kasai A. Ishikawa Y. (2014). Exendin-4 Ameliorates Cardiac Ischemia/reperfusion Injury via Caveolae and Caveolins-3. Cardiovasc Diabetol. 13, 132. 10.1186/s12933-014-0132-9 25194961 PMC4172825

[B52] UK Prospective Diabetes Study (UKPDS) Group (1998). Intensive Blood-Glucose Control with Sulphonylureas or Insulin Compared with Conventional Treatment and Risk of Complications in Patients with Type 2 Diabetes (UKPDS 33). UK Prospective Diabetes Study (UKPDS) Group. Lancet 352, 837–853. 9742976

[B53] Ussher J. R. Drucker D. J. (2014). Cardiovascular Actions of Incretin-Based Therapies. Circ. Res. 114, 1788–1803. 10.1161/circresaha.114.301958 24855202

[B54] Ussher J. R. Wang W. Gandhi M. Keung W. Samokhvalov V. Oka T. (2012). Stimulation of Glucose Oxidation Protects against Acute Myocardial Infarction and Reperfusion Injury. Cardiovasc Res. 94, 359–369. 10.1093/cvr/cvs129 22436846

[B55] Wiviott S. D. Raz I. Bonaca M. P. Mosenzon O. Kato E. T. Cahn A. (2019). Dapagliflozin and Cardiovascular Outcomes in Type 2 Diabetes. N. Engl. J. Med. 380, 347–357. 10.1056/nejmoa1812389 30415602

[B56] Woo J. S. Kim W. Ha S. J. Kim J. B. Kim S.-J. Kim W.-S. (2013). Cardioprotective Effects of Exenatide in Patients with ST-Segment-Elevation Myocardial Infarction Undergoing Primary Percutaneous Coronary Intervention. Atvb 33, 2252–2260. 10.1161/atvbaha.113.301586 23868944

[B57] Yu Y. W. Que J. Q. Liu S. Huang K. Y. Qian L. Weng Y. B. (2021). Sodium-Glucose Co-transporter-2 Inhibitor of Dapagliflozin Attenuates Myocardial Ischemia/Reperfusion Injury by Limiting NLRP3 Inflammasome Activation and Modulating Autophagy. Front. Cardiovasc Med. 8, 768214. 10.3389/fcvm.2021.768214 35083298 PMC8785320

[B58] Zinman B. Wanner C. Lachin J. M. Fitchett D. Bluhmki E. Hantel S. (2015). Empagliflozin, Cardiovascular Outcomes, and Mortality in Type 2 Diabetes. N. Engl. J. Med. 373, 2117–2128. 10.1056/nejmoa1504720 26378978

